# Two Cases of Compound Fracture of the Cranium with Depression

**Published:** 1888-10

**Authors:** W. D. Bizzell

**Affiliations:** Atlanta, Ga., Professor Principles and Practice of Medicine, Southern Medical College


THE ATLANTA MEDICAL AND SURGICAL JOURNAL.

VOL. V. OCTOBER, 1888. No. 8.

### Original Communications.

### TWO CASES OF COMPOUND FRACTURE OF THE CRANIUM WITH DEPRESSION.

BY W. D. BIZZELL, M. D., ATLANTA, GA., PROFESSOR PRINCIPLES AND PRACTICE OF MEDICINE, SOUTHERN MEDICAL COLLEGE.

I report these two cases to show the difference as to time and trouble necessary to a cure, where a thorough aseptic treatment is practiced and where it is neglected.

CASE I.—Lewis (c.), aged about 32 years. Saw the patient first on 16th December, 1887. History: three days before, a short piece of scantling had fallen endways from the top of a tall building in process of construction, striking the right parietal near the eminence, and about one inch to the right of the sagital suture. He was knocked senseless, and a wound in the scalp about two inches in length communicated with a depressed and comminuted fracture of the parietal about one and a half by seven-eighths inch. On recovering consciousness he did not complain much; had no signs of cerebral compression; walked to a drug-store, where the wound was sewed up.

When I saw him, three days afterward, was in bed, with temperature 102, complaining of throbbing pain in head. There was considerable swelling of the right side of the scalp and face, right eye partially closed, nervous, restless and unable to sleep. Removed the dressings and bandages, which had become soaked and putrid, removed the stitches and gave exit to sanious offensive pus. From a small, triangular opening, where two of the fragments of depressed bone approached the lower anterior angle of the wound, pus was seen to well up with each pulsation of the brain beneath.

On account of the fever, the septic condition of the wound and the fact that no signs of compression existed, I thought it best not to disturb the fragments. A fine drainage tube of rubber was carried down to this point and made to engage in the bony opening, but not allowed to penetrate into the cranium. The hair for a considerable area round the wound was cut away and the wound thoroughly washed with a four per cent. solution of carbolic acid in boiled water, dusted with iodoform, an iodoform pad of absorbent cotton applied and over this a large pad of borated cotton, all secured in place by adhesive straps. In forty-eight hours all of the disagreeable symptoms had subsided. If he lay for an hour or two in such position as to prevent a free drainage from the cranial wound, a severe, throbbing headache would be set up. He soon learned, however, to relieve this by turning in such position as to promote drainage. The suppuration was free and the wound was dressed daily for two weeks; after that it was dressed every second day. By the fourth week it had closed up to a fistulous opening the size of the drainage tube. The probe showed dead bone. Three weeks later this was removed; an exfoliation of the outer layer of cranium three-quarters of an inch by one-half inch; the wound then healed. One month later it was reported to me that he was unable to work, complaining of headache. Thought it might probably be due to a localized meningitis or to the growth of an osteophite. After a judicious questioning, became satisfied that it was due to the *irritating talk*

*of a damage-case lawyer.* The pain disappeared after a settlement with his employer.

CASE II.—Charlie F. (c.), aged 17 years. Was called evening June 4th, 1888, by Dr. J. S. Holliday, a retired physician, in whose yard the injury had been inflicted. Had been struck by a stone size of a turkey egg, thrown with great force by a twelve year old white boy, distant about ten feet. Those who saw him said that he did not fall, but spun round and seemed dazed. While Dr. Holliday was examining the extent of the injury, the boy suddenly lost consciousness and became convulsed. When I saw him, some twenty minutes after injury, was lying quiet, breathing stertorously and could not be roused.

Found a ragged wound of the scalp just within the edge of hair, about three-fourths inch in front of coronal suture of left side and on a line dropped vertically at junction of middle and outer third of orbit. There was a comminuted fracture, with considerable depression of the corresponding frontal bone. Securing the assistance of Dr. W. S. Elkin, who anæsthetized the patient, we shaved the scalp for a considerable distance from the wound, scrubbed it thoroughly with soap and water and washed the wound and skin with a four per cent. sol. carbolic acid. We then enlarged the scalp wound, hands and instruments being thoroughly aseptic; with the elevator raised and with forceps removed, four small pieces of bone and several small clots of blood, a small opening was then discovered in the dura through which the cerebro-spinal fluid flowed freely. Warm water recently boiled, containing one per cent. of carbolic acid, was ladled into the wound with a clean spoon until it welled out freely over the edges of the wound. It was then dusted with iodoform, three strands of aseptic silk laid in the bottom and brought out at the most dependent portion of the wound and closed with fine silk sutures. Over all a dry pad of iodoform absorbent cotton was placed and over this a large lot of borated cotton secured in place by bandage. A bladder of ice was placed over the dressing and kept there continuously. The drainage was moderate for the first twenty-four hours and then ceased. A careful examination showed that the dressings were odorless and drying out nicely.

They were not removed till fifth day, when the wound was found healed, except a small part in the centre where the tissues had been crushed by the stone. The drainage silk was removed. On the seventh day the sutures were all removed, and on the ninth day the patient was discharged.

Our patient was perfectly rational on recovery from the anæsthetic; though a little irritable for the first two days, never complained at all, and was up continuously after the third day, and, as I say, was dismissed on the ninth. The bare report of these cases carries its own lesson, and it is hardly necessary to comment thereon.

